# SNRPB2 facilitates esophageal squamous cell carcinoma oncogenesis and progression via E2F4 stabilization

**DOI:** 10.3389/fimmu.2025.1610721

**Published:** 2025-06-19

**Authors:** Feng Xu, Chen-Cheng Zhu, Chen Lu, Guang-Yao Ning, Ren-Quan Zhang

**Affiliations:** Department of Thoracic Surgery, the First Affiliated Hospital of Anhui Medical University, Hefei, Anhui, China

**Keywords:** SNRPB2, esophageal cancer, cancer biomarker, clinical relevance, prognostic factor, tumor progression, immune infiltration

## Abstract

**Introduction:**

Esophageal cancer (ESCA) is a highly aggressive malignancy with poor prognosis. Small nuclear ribonucleoprotein polypeptide B2 (SNRPB2) is a core component of the spliceosome involved in pre-mRNA splicing. However, its role in tumor development and progression remains largely unclear. This study aimed to evaluate the clinical relevance and prognostic value of SNRPB2 in ESCA.

**Methods:**

SNRPB2 mRNA expression and genetic alterations were analyzed using GEPIA2 and cBioPortal. Protein expression was assessed by immunohistochemistry in paraffin-embedded esophageal squamous cell carcinoma (ESCC) tissues. Functional assays in ESCC cell lines were conducted to determine the biological role of SNRPB2. Immune-related and functional analyses were performed using TIMER, TISIDB, TISCH, Gene Ontology (GO), and Gene Set Enrichment Analysis (GSEA). Cycloheximide (CHX) chase assays were used to assess protein stability.

**Results:**

SNRPB2 mRNA was upregulated in ESCA and associated with tumor progression and poor prognosis. Immunohistochemistry confirmed high SNRPB2 protein expression in ESCC, correlating with vessel carcinoma embolus, lymph node metastasis, clinical stage, and tumor grade. SNRPB2 knockdown significantly inhibited ESCC cell proliferation, migration, and invasion *in vitro* and *in vivo*. GSEA indicated that SNRPB2 suppresses the Rb/E2F pathway. Mechanistically, SNRPB2 stabilized E2F4 protein by preventing its proteasomal degradation, and E2F4 overexpression reversed the tumor-suppressive effects of SNRPB2 silencing. Immune analyses showed that SNRPB2 expression correlated with increased infiltration of activated CD8^+^ T cells, γδ T cells, dendritic cells, and monocytes, as well as immune-related genes including *PDCD1*, *CD274*, *CTLA4*, *HLA-DRA*, and *B2M*. These findings suggest a dual role for SNRPB2 in promoting tumor progression and modulating the immune microenvironment in ESCA.

**Conclusion:**

SNRPB2 promotes ESCC progression by stabilizing E2F4 and regulating cell cycle genes. It is also associated with immune infiltration and gene expression in ESCA. SNRPB2 may serve as a prognostic biomarker and potential therapeutic target in esophageal cancer.

## Introduction

Esophageal cancer (ESCA) is one of the most lethal malignancies (1). Surgical resection of the tumor from the primary site has been the standard treatment, especially for localized esophageal squamous cell carcinoma (ESCC), which is the main histological type of ESCA in China (2). Nowadays, proteogenomics and single-cell transcriptomic analyses have elucidated cancer-driving waves in ESCC progression and revealed the molecular characterization of dietary habit-associated signatures (3, 4). However, the intrinsic characteristics of the molecular and clinical perspectives associated with the risk factors of ESCC progression are still unknown. Therefore, further study of the comprehensive understanding of ESCC molecular targets will help overcome the current challenges in precision therapeutics and improve outcomes for ESCC patients.

Small nuclear ribonucleoprotein polypeptide B2 (SNRPB2), known as U2 snRNP B’’ or U2 small nuclear ribonucleoprotein B’’, is one of the unique proteins that comprise the U2 snRNP (5). SNRPB2 has unique RNA binding property, with the high degree of sequence and structural conservation (6), involved in precursor messenger RNA (pre-mRNA) splicing as component of the spliceosome in physiological state (7, 8). Pre-mRNA splicing are key for eukaryotic gene expression and cellular function while splicing alterations can lead to various diseases including blindness (9), autoimmunity disease and cancer (10, 11). In tumors, alternative splicing frequently plays critical roles in tumor genesis, development and metastasis, whose regulatory factors may be considered as prognostic biomarkers and therapeutic targets for cancer intervention (12). SNRPB2 as an early growth-inducible gene essential for the regulation of pre-mRNA splicing and gene expression, is widely expressed in primary cortical neurons, epithelial cells and fibroblasts (13, 14). However, the potential role of SNRPB2 in tumors remain ill-defined, necessitating further evaluation.

In this study, we identified SNRPB2 as a previously underappreciated oncogenic factor in ESCC. We found that SNRPB2 was significantly upregulated in ESCC tissues and closely associated with adverse clinicopathological features and poor prognosis. Functional assays revealed that SNRPB2 promotes ESCC cell proliferation, migration, and invasion both *in vitro* and *in vivo*. Mechanistically, SNRPB2 stabilized the transcriptional repressor E2F4 by inhibiting its proteasomal degradation, thereby regulating downstream cell cycl-related genes. In addition, immune-related analyses revealed that SNRPB2 expression was positively correlated with immune cell infiltration and the expression of immunoregulatory genes, suggesting a potential role in modulating the tumor immune microenvironment. These findings highlight SNRPB2 as a promising prognostic biomarker and potential therapeutic target in ESCC.

## Materials and methods

### Cell lines and lentivirus-mediated gene knockdown

The ESCC cell lines (KYSE-150, KYSE-140, KYSE-450, and TE-1) and the human immortalized esophageal epithelial cell line (NE-1) were purchased from the Cell Bank of the Chinese Academy (Shanghai, China). All ESCC cells were cultured in RPMI-1640 supplemented with 10% FBS and 100IU/ml Penicillin-Streptomycin solution with 5% CO_2_ at 37°C.

For lentiviral gene knockdown, a pLKO.1 shRNA was generated against human SNRPB2 target sequences. pLKO lentiviral vectors containing shRNA were transfected into 293T cells together with psPAX2 and pMD2.G by calcium phosphate transfection (Sigma-Aldrich). The shRNA sequences were transfected into the TE-1 and KYSE-150 cells.

shSNRPB2#1: Forward:5’-CCGGCCCAAGGAAATTCAACACCAACTCGAGTTGGTGTTGAATTTCCTTGGGTTTTT-3’
Reverse: 5’-AATTCAAAAACCCAAGGAAATTCAACACCAACTCGAGTTGGTGTTGAATTTCCTTGGG-3’
shSNRPB2#2: Forward:5’-CCGGCCATGCTATGAAGATCACCTACTCGAGTAGGTGATCTTCATAGCATGGTTTTT-3’
Reverse: 5’-AATTCAAAAACCATGCTATGAAGATCACCTACTCGAGTAGGTGATCTTCATAGCATGG-3’
shE2F4#1:
Forward: 5’-CCGGCCCTCTCTTCATTTCGGCTTTCTCGAGAAAGCCGAAATGAAGAGAGGGTTTTT-3’
Reverse: 5’-AATTCAAAAACCCTCTCTTCATTTCGGCTTTCTCGAGAAAGCCGAAATGAAGAGAGGG-3’
shE2F4#2:
Forward: 5’-CCGGCGGATTTACGACATTACCAATCTCGAGATTGGTAATGTCGTAAATCCGTTTTT-3’
Reverse: 5’-AATTCAAAAACGGATTTACGACATTACCAATCTCGAGATTGGTAATGTCGTAAATCCG-3’

### Patient tissue sample collection

One hundred and twenty-five paraffin-embedded ESCC tissues and paired normal esophageal epithelial tissues adjacent to the cancer tissue were collected from patients who underwent surgery at the First Affiliated Hospital of Anhui Medical University (Hefei, Anhui, China) between 2015 and 2018. None of the patients had a history of other malignant tumors or had received preoperative interventions such as radiotherapy or chemotherapy. Each patient provided written informed consent, and the study was approved by the Clinical Research Ethics Committee of The First Affiliated Hospital of Anhui Medical University (Approval No. AHMU-FAH-EC-PJ2025-01-81).

### GEPIA2, cBioPortal analysis

A correlative prognostic analysis of SNRPB2 from the GEPIA2 dataset, including overall survival (OS), disease-specific survival (DSS), and progression-free interval (PFI), was conducted using the log-rank test at a median cut-off of 50% for both the SNRPB2^low^ and SNRPB2^high^ cohorts. The cBioPortal for Cancer Genomics (http://cbioportal.org) served as an open online resource for visualizing multidimensional cancer genomics datasets (15).

### TIMER, TISIDB, and TISCH analysis

To assess the immune landscape associated with SNRPB2, the TIMER database (https://cistrome.shinyapps.io/timer/) was used to analyze correlations between SNRPB2 expression and infiltration levels of major immune cell types, including B cells, CD8^+^ T cells, CD4^+^ T cells, macrophages, neutrophils, and dendritic cells (16). The TISIDB platform was utilized to explore the relationships between SNRPB2 and a range of immune modulators, such as immunoinhibitory and immunostimulatory factors, MHC molecules, and chemokines across various cancer types. Additionally, single-cell transcriptomic data from TISCH provided insights into SNRPB2 expression across immune and stromal cell subsets within the ESCA tumor microenvironment.

### GO and GSEA enrichment analysis

Functional enrichment analysis was performed using the R package clusterProfiler to explore the biological functions of SNRPB2 in ESCA. Gene Ontology (GO) and Gene Set Enrichment Analysis (GSEA) were applied for pathway-level annotation. For GO analysis, pathways with *P* < 0.05 and adjusted *P* < 0.05 were considered statistically significant. For GSEA, gene sets with a false discovery rate (FDR) < 0.05 were regarded as significantly enriched. Adjusted *P*-values were calculated using the Benjamini-Hochberg method.

### Xenograft tumor model assay

Four-week-old male athymic BALB/c nude mice were purchased from Weitonglihua Co., Ltd. (China) and housed in a specific pathogen-free (SPF) facility under standard conditions (12-hour light/dark cycle, 22 ± 2°C temperature, 55 ± 10% humidity), with ad libitum access to food and water. Environmental enrichment was provided. Twenty mice were randomly assigned (n = 5/group) into four groups: (1) shCtrl + Vector, (2) shSNRPB2 + Vector, (3) shCtrl + E2F4, and (4) shSNRPB2 + E2F4. Group sizes were based on preliminary data and power analysis. TE-1 cells (3 × 10^6^ in 200 μL PBS) were injected subcutaneously into the right axillary region. Anesthesia was induced and maintained with isoflurane (2–3% induction, 1.5–2% maintenance). Depth of anesthesia was confirmed by loss of reflexes and slowed respiration. Tumor volume was measured every three days using calipers and calculated as 0.5 × length × width². On day 21, mice were euthanized via CO_2_ inhalation (30–50% chamber volume/min), followed by cervical dislocation to confirm death. Tumors were harvested, photographed, and weighed. Predefined humane endpoints included >20% body weight loss, tumor diameter >2 cm, skin ulceration, or signs of severe distress. No animals met these criteria before study termination. No animals or data points were excluded. Randomization was performed using a random number table. Blinding was applied during tumor measurement and data analysis but not during treatment administration. Tumor volume was the primary endpoint; tumor weight and imaging served as secondary outcomes. All procedures were approved by the Laboratory Animal Ethics Committee of Anhui Medical University (Approval No. LLSC20242529) and conducted in accordance with institutional guidelines.

### Immunohistochemical analysis

The *in situ* protein expression levels of SNRPB2 and E2F4 in paraffin-embedded ESCC tissue sections were assessed by immunohistochemistry using rabbit polyclonal antibodies against SNRPB2 (1:100, 21244-1-AP, Proteintech) and E2F4 (1:100, 10923-1-AP, Proteintech). Five fields were randomly observed at a high magnification under a microscope. The staining intensity of SNRPB2 and E2F4 in tumor cells was classified as follows: 0 (no staining); 1 (light yellow); 2 (medium yellow); and 3 (dark yellow). The percentage of stained cells was categorized as follows: 0 (no positive tumor cells), 1 (<20% positive cells), 2 (20%-60% positive cells), and 3 (>60% positive cells). The total score, which ranged from 0 to 6, was calculated by summing the two scores. Samples with staining scores of 0–3 were classified as having low SNRPB2 or E2F4 expression, whereas those with scores >3 were classified as having high SNRPB2 or E2F4 (17).

### Western blot assay

Whole cell lysates were prepared using radioimmunoprecipitation assay (RIPA) lysis buffer (Beyotime) containing a complete protease inhibitor cocktail. The total protein was loaded and separated by SDS-PAGE, followed by transfer onto polyvinylidene fluoride membranes (Millipore, USA). Immunodetection was performed using antibodies against β-actin (1:5000, 81115-1-RR; Proteintech) and SNRPB2 (1:2000). Horseradish peroxidase-conjugated secondary antibodies (Cell Signaling Technology) were used for digital chemiluminescence detection (GE Healthcare). Blots are representative of three independent experiments.

### Cell proliferation assay

Cell proliferation was assessed using cell counting kit‐8 (CCK‐8) description (Beyotime). Cells (5×10^3^ cells/well) in logarithmic growth phase were seeded in 96-well plates and incubated at 37°C. After 24, 48, 72, 96 h and 120h, 10μL of CCK8 was added to each well and incubated for 2h. Cell proliferation was determined by measuring the absorption value of whole wells, which was directly detected at 450 nm.

### Clone formation assay

The cells were inoculated into six-well plates (1,000 cells/well) in triplicate and cultured for 14 days. Cells number more than 50 or cell size between 0.3 and 1.0 mm were considered as a single clone. All colonies were fixed with 4% paraformaldehyde for 10  min, stained with Giemsa, and photographed using a digital camera.

### Wound healing assay

The cells were collected and inoculated into six‐well plates (5×10^5^ cells/well) in 2 ml medium and cultured at 37°C with 5% CO_2_. When the cells reached more than 90% confluence, a pipetting tip of 100μL was used to scratch the six-well plate vertically. Cells were washed twice with PBS and cultured in an incubator with 5% CO_2_ at 37°C. Photographs were taken at 0 and 48 h under a microscope, and the experiment was repeated thrice.

### Transwell assay

The migration and invasion capacities of ESCC cells were determined using a Transwell chamber (Corning) precoated with Matrigel. After transfection, ESCC cells were collected, counted, and incubated in the upper chamber with 100μL RPMI-1640 medium without FBS in a 24-well plate (5×10^4^ cells/well). 600μL medium supplemented with 20% FBS was added to the lower chamber. After incubation at 37°C and 5% CO_2_ for 24 h, non-metastatic cells were removed using a cotton swab. 500μL Giemsa was added for staining and the migratory ability of the cells was analyzed. Representative images were selected from three independent experiments, of which five fields per chamber were randomly selected to count cell numbers. The magnification used was 200× .

### Co-immunoprecipitation

The cell extracts were resuspended and lysed on ice using an IP buffer. Protein A/G magnetic beads (Bimake, China) were added to cell lysates and incubated for 2 h at 4°C. IgG was added to the negative control group, whereas IP antibody was added to the experimental group for 12 h at 4°C. Immunoprecipitated proteins were eluted by boiling in SDS loading buffer (2×) for western blot analysis as described above.

### Cycloheximide chase assay

Cells were seeded in six-well plates (1×10^5^ cells/well) and pretreated with CHX (100 μg/mL) for 0–4 h. Cells were harvested for western blot analysis, as described above.

### Statistical analysis

SPSS (version 22.0) was used for data analysis. Chi-square test and paired-samples t-test were used for variable comparison, with *P* < 0.05 regarded as statistically significant. The normality of data distribution was assessed using the Shapiro-Wilk test. If data did not meet normal distribution assumptions, non-parametric alternatives such as the Mann-Whitney U test were considered.

## Results

### High expression of SNRPB2 mRNA was correlated with worse outcome in patients with ESCA

**Figure 1:**
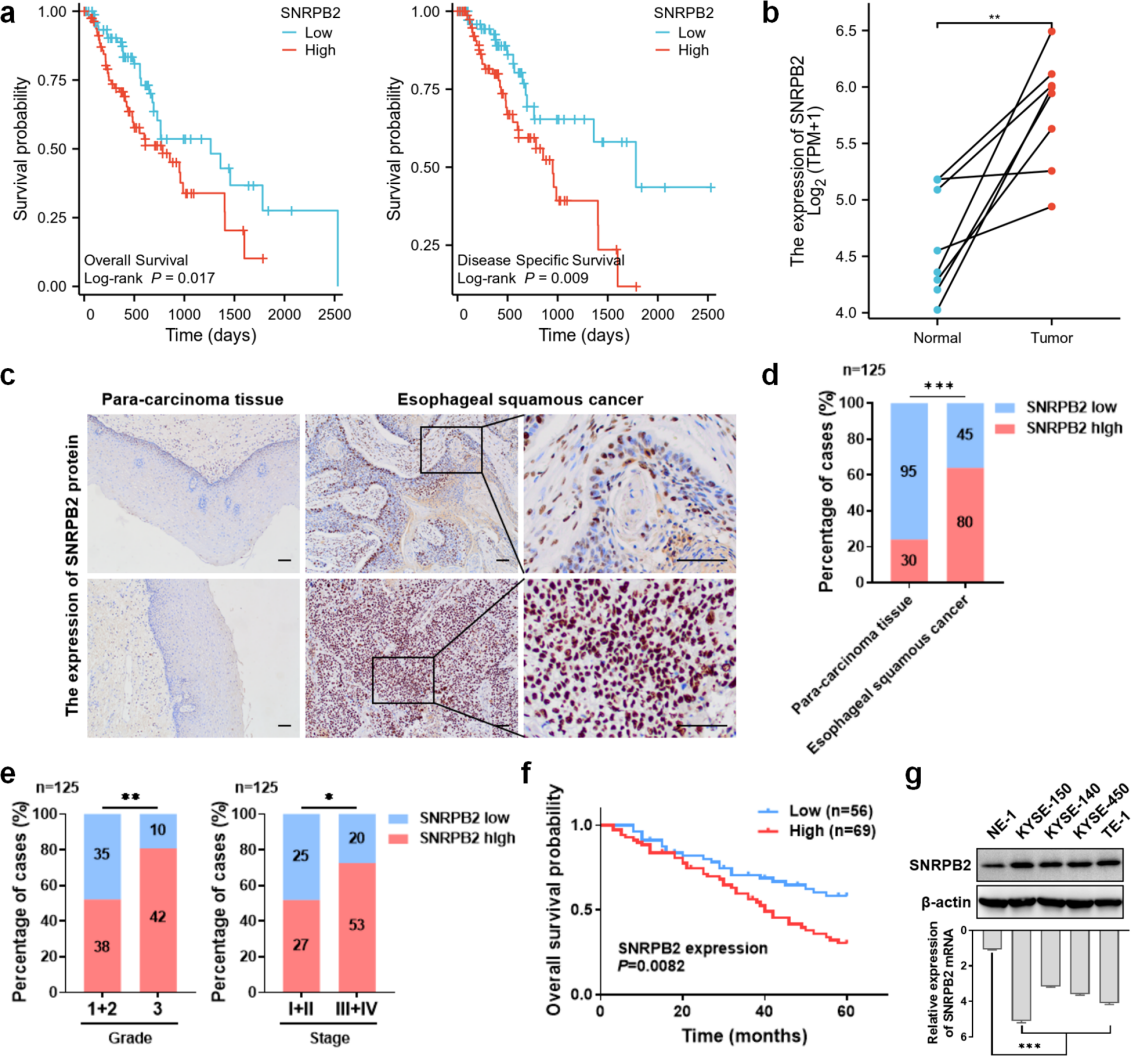
SNRPB2 was highly expressed in ESCA which was associated with unfavorable clinical characteristics and poor prognosis. **(a)** Kaplan-Meier curves were used to analyze the influence of SNRPB2 mRNA on OS and DSS in ESCA patients. **(b)** Relative SNRPB2 mRNA expression in paired normal and tumor tissues from ESCA patients. **(c)** Representative IHC images of SNRPB2 protein expression in tumor and para-carcinoma tissues of ESCC. The upper panel shows low-grade ESCC and the lower panel high-grade ESCC, each with para-carcinoma tissue and tumor samples. Scale bars: 100 μm. **(d)** The expression levels of SNRPB2 protein in para-carcinoma tissues versus ESCC tissues. **(e)** Association of SNRPB2 protein expression with tumor grade and stage in ESCC. **(f)** The correlation between SNRPB2 protein expression and OS of ESCC. **(g)** Protein and relative mRNA levels of SNRPB2 in normal esophageal epithelial cells and ESCC cell lines were determined by western blot and q-PCR, with β-actin as the loading control. Data represent mean ± SD from three independent experiments. Statistical analysis was performed using Student’s t-test and two-sided t-test. (*P < 0.05; **P < 0.01; ***P < 0.001).

Differentially expressed genes were identified by comparing ESCA and para-carcinoma esophageal tissue samples from the GEPIA2 database. We found that SNRPB2 mRNA was significantly upregulated in esophageal cancer significantly and correlated with the clinical stage and prognosis (**Supplementary Material Tables S1**, **S2**). Moreover, SNRPB2 expression was significantly higher in tumor tissues compared to matched normal tissues across multiple cancer types (**Supplementary Figure S1a**). To further determine the prognostic relevance of SNRPB2 in ESCA, patients from multiple ESCA datasets were classified into SNRPB2^high^ and SNRPB2^low^ groups and their survival (including OS, DSS, and PFI) was analyzed using Kaplan-Meier curves. The SNRPB2^low^ group had significantly longer OS and DSS than the SNRPB2^high^ group (both *P* < 0.05, **Figure 1a**), while the PFI showed no difference in this dataset (*P* > 0.05, **Supplementary Figure S1b**). These findings suggest that SNRPB2 mRNA expression might correlate with ESCA prognosis, whereas SNRPB2 overexpression might indicate worse outcomes in patients with ESCA.

To explore the expression of SNRPB2 mRNA in ESCA and normal esophageal tissue samples from TCGA, we analyzed para-carcinoma tissue from patients with ESCA and normal esophageal tissue from tumor-free subjects separately. We found that SNRPB2 mRNA was highly expressed in ESCA samples compared to normal samples, and similar results were observed in paired samples (both *P* < 0.05, **Supplementary Figure S1c**; **Figure 1b**), while no significant correlation between SNRPB2 mRNA expression and tumor stage was observed in this dataset (*P* > 0.05, **Supplementary Figure S1d**).

### Expression level of SNRPB2 protein in ESCC

To further validate the above results, we performed immunohistochemical staining of paraffin-embedded tissue specimens to detect the expression of SNRPB2 protein in ESCC. SNRPB2 protein is located in the nucleus and is extensively expressed in normal squamous epithelial cells and ESCC (**Figure 1c**). The expression rate of SNRPB2 in ESCC was 64.0%, which was significantly higher than that in adjacent tissues (24.0%, *P* < 0.05) (**Figure 1d**, **Table 1**). The results showed that SNRPB2 protein was significantly highly expressed in ESCC samples compared to paired normal samples, whose expression trends were in consistent with the findings obtained from TCGA.

**Table 1:** The expression levels of SNRPB2 protein in tumor and para-carcinoma tissues of patients with esophageal squamous cell carcinoma.

Parameter	n	SNRPB2		P Value
		Low expression	High expression	
Para-carcinoma tissue	125	95 (76.0%)	30 (24.0%)	**<0.001**
Esophageal squamous cancer	125	45 (36.0%)	80 (64.0%)	

Bold values indicate statistically significant differences (P < 0.05).

### Association between the expression of SNRPB2 protein and the clinicopathological characteristics of patients with ESCC

To explore the clinical significance of SNRPB2 expression in ESCC, we correlated its expression with the clinicopathological characteristics of ESCC patients. Interestingly, the expression levels of SNRPB2 protein were positively correlated with high tumor grade and clinical stage (both *P* < 0.05) (**Table 2**, **Figure 1e**). Furthermore, the expression levels of SNRPB2 protein in ESCC were significantly correlated with vessel carcinoma embolus and lymph node metastasis, (both *P* < 0.05) but not with patient age, sex, BMI, smoking or drinking history, tumor location, or nerve invasion (all *P* > 0.05) (**Table 2**; **Supplementary Figure S1e**). Kaplan-Meier analysis showed that ESCC patients with SNRPB2 high expression had significantly poorer overall survival compared with SNRPB2^low^ group (*P* < 0.05) (**Figure 1f**). Collectively, the expression of SNRPB2 may be associated with ESCC progression, which is associated with poor prognosis in ESCC.

**Table 2:** The relationship between SNRPB2 expression and the clinicopathological features of esophageal squamous cell carcinoma.

Parameter	n	SNRPB2		*P* Value
		Low expression	High expression	
Age (years)				0.601
≤60	30	12 (40.0%)	18 (60.0%)	
>60	95	33 (34.7%)	62 (65.3%)	
Sex				0.824
male	93	34 (36.6%)	59 (63.4%)	
female	32	11 (34.4%)	21 (65.6%)	
Smoking				0.920
Yes	34	12 (35.3%)	22 (64.7%)	
No	91	33 (36.3%)	58 (63.7%)	
Drinking				0.781
Yes	37	14 (37.8%)	23 (62.2%)	
No	88	31 (35.2%)	57 (64.8%)	
BMI				0.840
≤28	113	41 (36.3%)	72 (63.7%)	
>28	12	4 (33.3%)	8 (66.7%)	
Grade				**0.001**
1 + 2	73	35 (47.9%)	38 (52.1%)	
3	52	10 (19.2%)	42 (80.8%)	
Vessel carcinoma embolus				**0.012**
+	66	17 (25.8%)	49 (74.2%)	
–	59	28 (47.5%)	31 (52.5%)	
Nerve invasion				0.068
+	58	16 (27.6%)	42 (72.4%)	
–	67	29 (43.3%)	38 (56.7%)	
Lymph node metastasis				**<0.001**
+	80	19 (23.7%)	61 (76.3%)	
–	45	26 (57.8%)	19 (42.2%)	
Stage				**0.018**
I+II	52	25 (48.1%)	27 (51.9%)	
III+IV	73	20 (27.4%)	53 (72.6%)	
Tumor location				0.507
Upper+Lower	35	11 (31.4%)	24 (68.6%)	
Middle	90	34 (37.8%)	56 (62.2%)	

Bold values indicate statistically significant differences (P < 0.05).

### SNRPB2 facilitated ESCC progression

To investigate the potential role of SNRPB2 in ESCC, we first determined SNRPB2 expression in various ESCC cells and human immortalized esophageal epithelial cells.

As expected, the protein and mRNA levels in NE-1 cells were markedly lower than those in tumor cells (**Figure 1g**).

**Figure 2:**
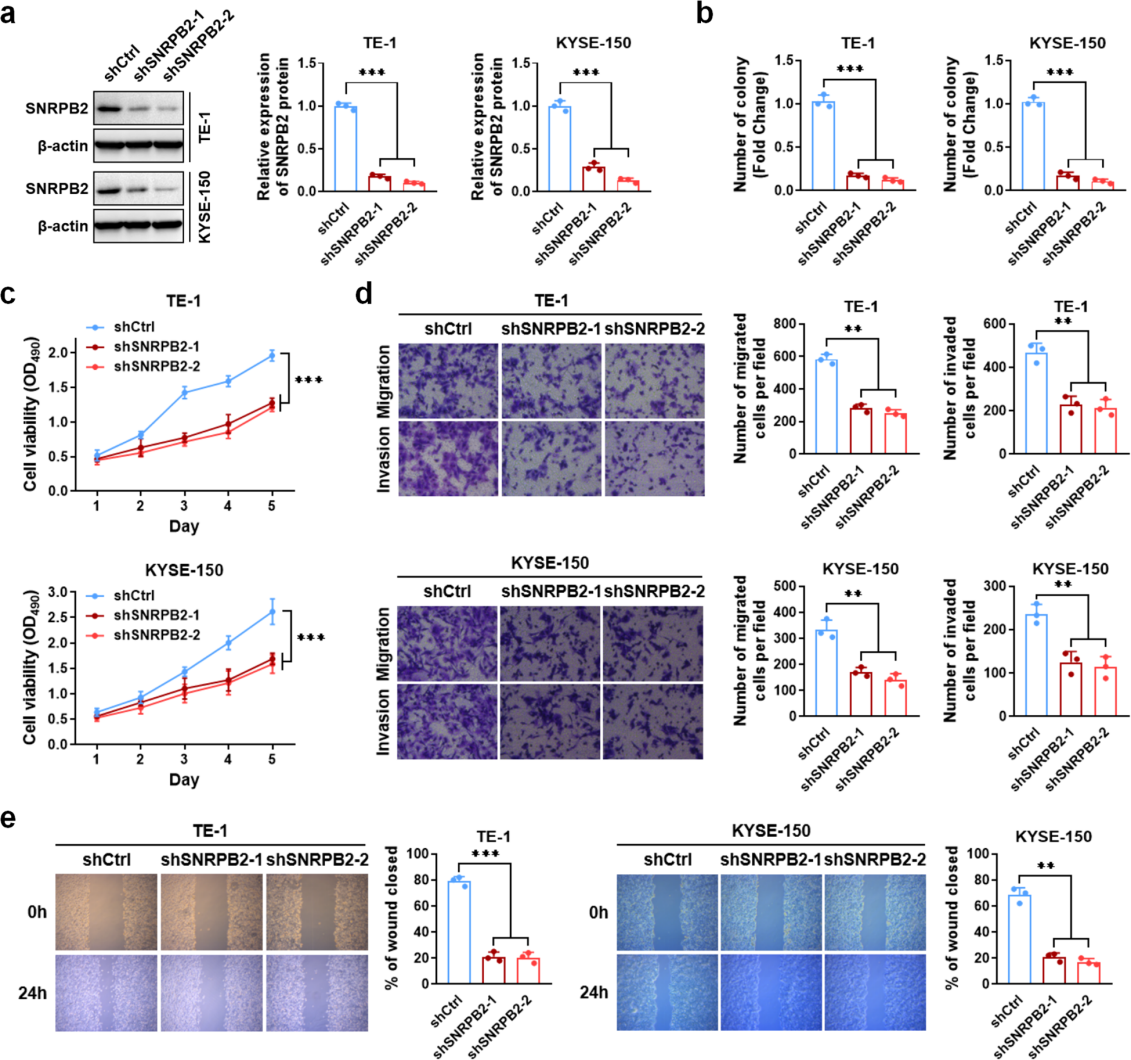
SNRPB2 promoted proliferation, migration and invasion of ESCC cells. **(a)** Western blot analysis of SNRPB2 protein levels in TE-1 and KYSE-150 cells after transduction with two independent shRNAs targeting SNRPB2, with β-actin as the loading control. Bar graphs on the right show quantification of relative SNRPB2 protein levels normalized to β-actin. **(b)** The number of colony cells decreased significantly when cells were treated with SNRPB2 shRNAs. **(c)** The cell viability significantly decreased when cells were treated with SNRPB2 shRNAs. **(d, e)** TE-1 and KYSE-150 cells transfected with SNRPB2 shRNAs were evaluated by transwell migration assay, matrigel invasion assay, and wound healing experiment. All data were presented as the mean ± SDs (n = 3). Statistical analysis was performed using Student’s t-test. (**P < 0.01; ***P < 0.001).

Based on the expression levels, SNRPB2 was knocked down in the TE-1 and KYSE-150 cells. Western blot analysis suggested that shSNRPB2#2 significantly inhibited the protein expression of SNRPB2, which was used for subsequent experiments (**Figure 2a**). Colony formation and cell proliferation assays indicated that knockdown SNRPB2 remarkably suppressed cell proliferation (**Figures 2b, c**; **Supplementary Figure S1f**). Transwell assays indicated that the expression of SNRPB2 was related with the migration and invasion of ESCC cells (**Figure 2d**). Wound healing assays indicated that the migration rates of TE-1 and KYSE-150 cells with SNRPB2#2 knockdown were 21% and 16%, respectively, lower than those of the control group (80% and 70%, respectively), suggesting that SNRPB2 promoted the migration of ESCC cells (**Figure 2e**). These results imply that SNRPB2 acts as an oncogene and facilitates ESCC progression.

### Genetic alterations and immunological correlation of SNRPB2 in ESCA

We conducted an online database analysis to investigate genetic alterations in SNRPB2 that may be associated with tumorigenesis via cBioPortal. Genetic alterations were detected in 1.1% of SNRPB2 genes in ESCA, including 5 amplifications, 2 deep deletions, 1 truncating mutation, and 1 missense mutation (**Supplementary Figure S2a**). No alterations in SNRPB2 were detected in ESCC based on an analysis of 227 samples from two studies (**Supplementary Figure S2b**). These results suggested that SNRPB2 is structurally conserved in ESCC cells.

### Immunological associations of SNRPB2 with immune regulatory networks in ESCA

**Figure 3:**
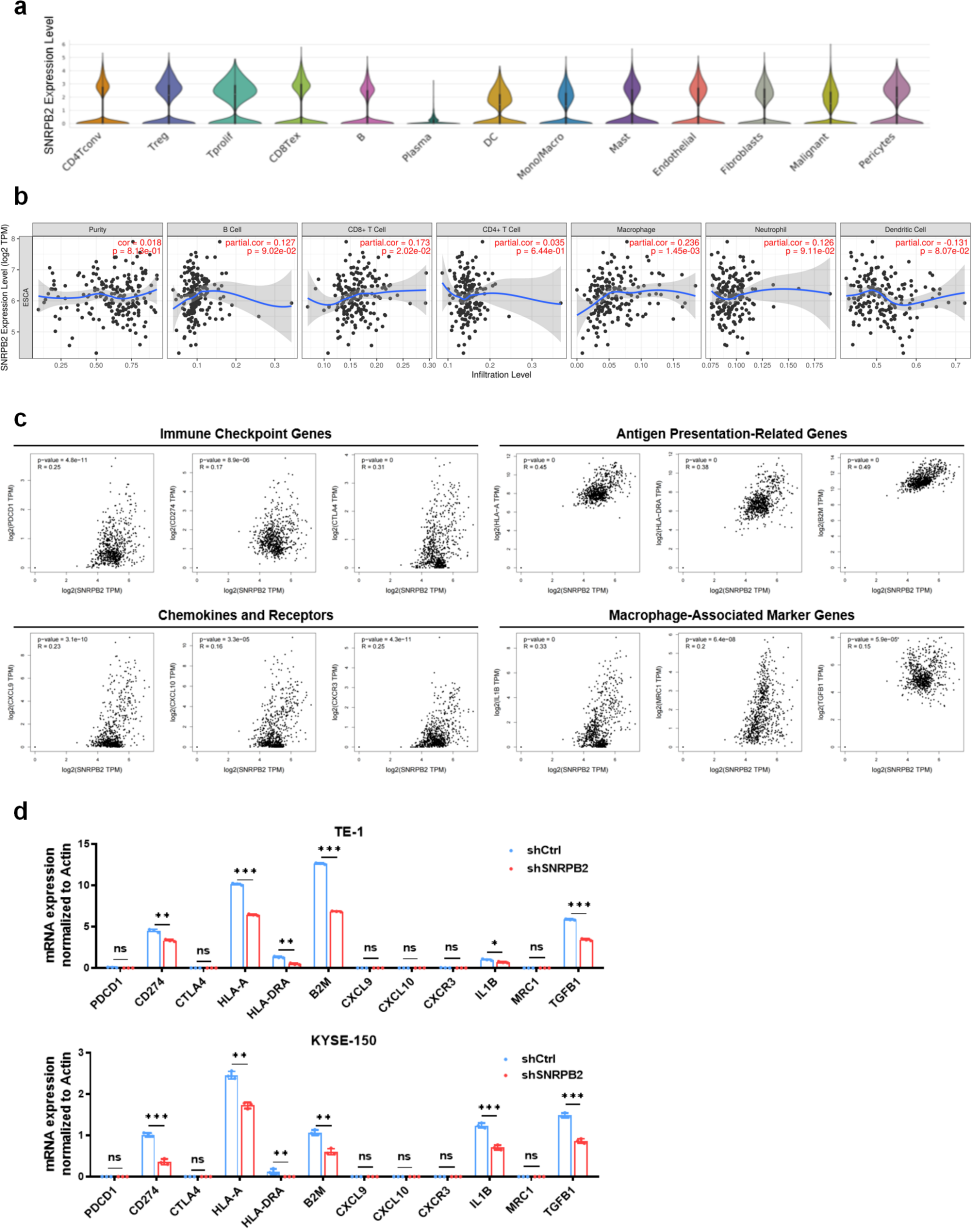
SNRPB2 Expression Is Associated with Immune Infiltration and Immune Gene Signatures in ESCC. **(a)** Single-cell expression distribution of SNRPB2 across different cell types in ESCA, based on dataset GSE160269. **(b)** Correlation between SNRPB2 expression and immune cell infiltration levels in ESCA using the TIMER database. **(c)** Correlation between SNRPB2 expression and representative immune-related genes from GEPIA2. **(d)** Relative mRNA expression levels of immune-related genes in TE-1 and KYSE-150 cells after SNRPB2 knockdown, analyzed by qPCR. Data represent mean ± SD from three independent experiments. Statistical analysis was performed using Student’s t-test. (*P < 0.05; **P < 0.01; ***P < 0.001; ns, not significant).

To investigate the potential immunological relevance of SNRPB2 in ESCA, we analyzed its single-cell expression profiles using dataset GSE160269. SNRPB2 expression was found to be relatively enriched in specific immune cell subsets, including proliferating T cells (Tprolif), exhausted CD8^+^ T cells (CD8Tex), dendritic cells (DC), and monocytes/macrophages (Mono/Macro) (**Figure 3a**). These findings suggest that SNRPB2 may play a role in modulating immune effector functions within the ESCA tumor microenvironment. To further validate this observation at the bulk transcriptomic level, we performed immune infiltration analysis using TIMER2.0. SNRPB2 expression showed a statistically significant, albeit modest, positive correlation with the infiltration levels of CD8^+^ T cells (partial cor = 0.173, *P* = 0.0202) and macrophages (partial cor = 0.236, *P* = 0.00145) in ESCA (**Figure 3b**), supporting its potential involvement in shaping the immune landscape.

To comprehensively evaluate the immune-related characteristics of SNRPB2 in ESCA, we conducted a pan-cancer analysis focusing on immune cell infiltration and functional immune signatures. As shown in **Supplementary Figure S3**, SNRPB2 expression in ESCA exhibited a positive correlation with the infiltration of multiple immune cell subsets, including activated CD8^+^ T cells, γδ T cells, activated dendritic cells, and monocytes (**Supplementary Figure S3a**). It also showed a moderate association with selected immunostimulatory genes such as PVR (**Supplementary Figure S3b**), whereas correlations with most immune inhibitory genes were minimal (**Supplementary Figure S3c**). In addition, SNRPB2 expression was positively linked to several MHC class I and II molecules, including *B2M*, *HLA-C*, and *HLA-DQA1* (**Supplementary Figure S3d**), as well as a subset of chemokines such as *CCL3*, *CCL4*, *CXCL8*, and *CXCL16* (**Supplementary Figure S3e**). In contrast, associations with chemokine receptors were weak or absent (**Supplementary Figure S3f**). To further support these findings, we examined the expression correlation of SNRPB2 with representative immune signatures in ESCA. As illustrated in **Figure 3c**, SNRPB2 expression was positively associated with immune checkpoint genes (*PDCD1*, *CD274*, *CTLA4*), antigen presentation-related genes (*HLA-A*, *HLA-DRA*, *B2M*), chemokines (*CXCL9*, *CXCL10*, *CXCR3*), and macrophage-associated markers (*IL1B*, *MRC1*, *TGFB1*), suggesting its potential role in modulating antitumor immune responses.

To validate the transcriptomic findings, we performed qPCR analysis in two ESCA cell lines. Knockdown of SNRPB2 in TE-1 and KYSE-150 cells led to significantly decreased expression of multiple immune-related genes, including *CD274*, *HLA-A*, *HLA-DRA*, *B2M*, *IL1B*, and *TGFB1* (**Figure 3d**). These results further support the immunomodulatory role of SNRPB2 in ESCA, highlighting its involvement in regulating antigen presentation, inflammatory signaling, and immune effector pathways. Together, these findings demonstrate that SNRPB2 plays an active role in shaping the immune landscape of ESCA and may represent a novel immune-regulatory target for therapeutic intervention.

### SNRPB2 inhibited Rb/E2F pathway in ESCA via increasing stability of E2F4 protein

**Figure 4:**
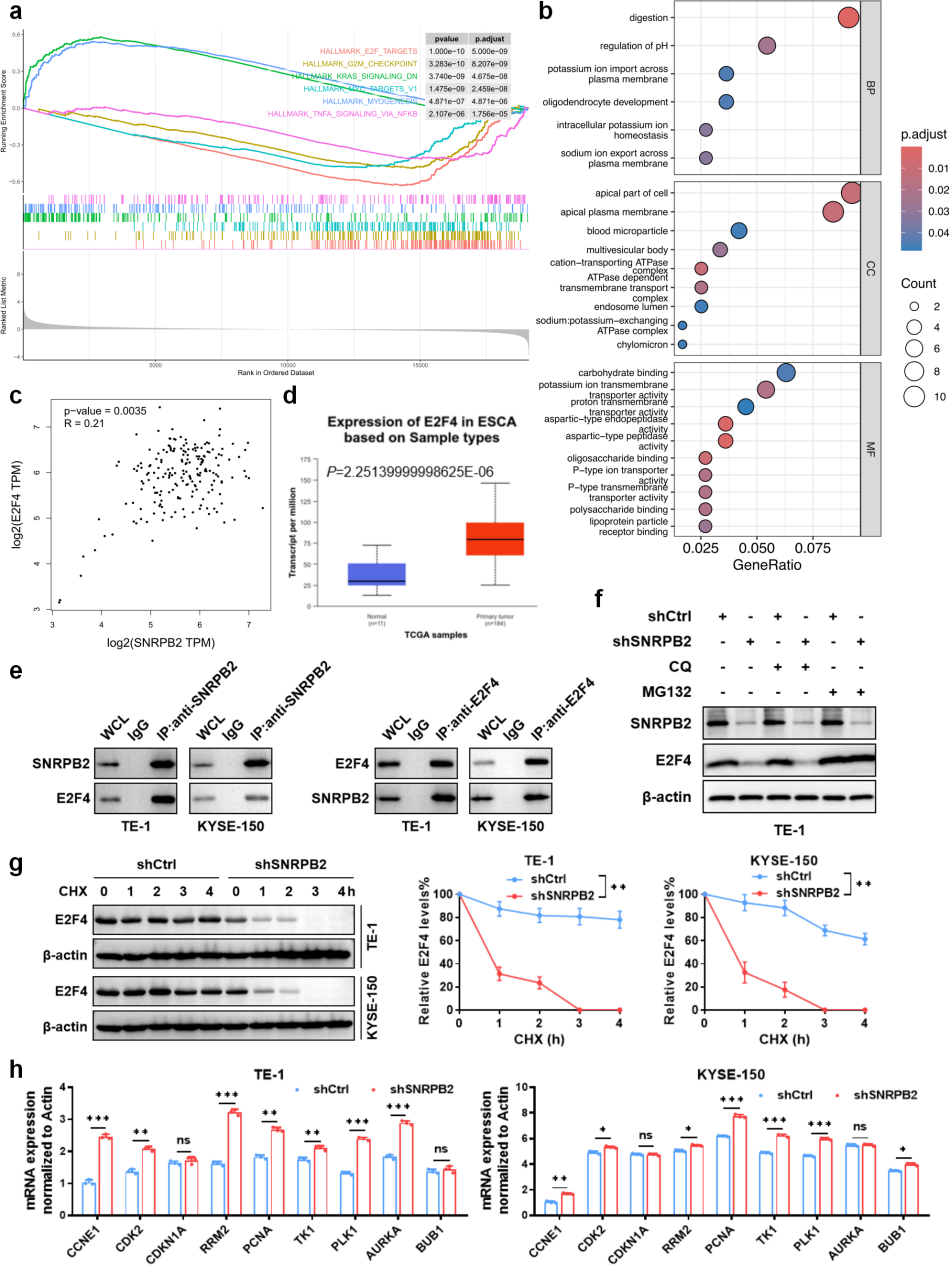
SNRPB2 inhibited Rb/E2F pathway in ESCA and interacted with E2F4 in ESCC cells. **(a)** Pathway enrichment: GSEA enrichment analyses of co‐expressed genes indicating an association of SNRPB2 with the top six signaling pathways in the TCGA-ESCA cohort. **(b)** GO functional enrichment analysis of SNRPB2 and its interactors were performed. **(c)** Correlation of the mRNA levels of SNRPB2 and E2F4 in TCGA-ESCA tumor samples. **(d)** The expression level of E2F4 mRNA in ESCA and normal esophageal tissue samples. **(e)** The Co-IP assay demonstrated the specific interactions between SNRPB2 and E2F4 in TE-1 and KYSE-150 cells. IgG was used as a negative control; whole cell lysates served as input. **(f)** Western blot analysis of E2F4 and SNRPB2 protein levels after treatment with MG132 or CQ in shCtrl or shSNRPB2 TE-1 cells. β-actin was used as the loading control. **(g)** The protein level of E2F4 after treated with CHX was measured by western blot in TE-1 and KYSE-150 cells, with β-actin as the internal control. Quantification of E2F4 band intensity normalized to β-actin is shown (right). **(h)** qPCR analysis of E2F4 target gene expression in TE-1 and KYSE-150 cells with or without SNRPB2 knockdown. β-actin was used for normalization. Data are represented as the mean ± SD (n = 3). Statistical analysis was performed using Student’s t-test. (*P < 0.05; **P < 0.01; ***P < 0.001; ns, not significant).

GO and GSEA analyses were performed to reveal the promoting effect of SNRPB2 on ESCC cells. The pathway enrichment results suggested that SNRPB2 significantly inhibited the Rb/E2F pathway in ESCA and was involved in the process of digestion in the apical part of the cell (**Figures 4a, b**). Furthermore, bioinformatic analysis indicated that the mRNA expression of SNRPB2 was positively correlated with E2F4, which is known as the transcription inhibitory factor of the Rb/E2F pathway (**Figure 4c**). As shown in **Figure 4d**, consistent with SNRPB2, the mRNA expression level of E2F4 was significantly upregulated in ESCA. To verify the interactions between SNRPB2 and E2F4, a co-immunoprecipitation assay was performed to confirm their interaction in TE-1 and KYSE-150 cells. The results of the co-IP assay demonstrated that SNRPB2 interacts with E2F4 (**Figure 4e**).

To further elucidate the mechanism by which SNRPB2 regulates E2F4, we evaluated the impact of SNRPB2 silencing on E2F4 protein stability. As shown in **Figure 4f**, treatment with the proteasome inhibitor MG132, but not the lysosomal inhibitor chloroquine (CQ), partially restored E2F4 levels in SNRPB2-knockdown TE-1 cells, suggesting that SNRPB2 may prevent E2F4 degradation via a proteasome-dependent pathway. Consistently, cycloheximide (CHX) chase assays demonstrated that E2F4 protein was more rapidly degraded in SNRPB2-deficient cells compared to controls, confirming that SNRPB2 contributes to the post-translational stabilization of E2F4 (**Figure 4g**). We next examined the expression of E2F4 downstream targets involved in cell cycle regulation. As shown in **Figure 4h**, knockdown of SNRPB2 significantly increased the mRNA levels of several E2F4-responsive genes, including *CCNE1*, *PCNA*, *TK1*, and *PLK1* in both TE-1 and KYSE-150 cells, suggesting that loss of E2F4 repression due to SNRPB2 silencing leads to transcriptional de-repression of cell cycle-promoting genes.

To support these findings, we assessed E2F4 mRNA and protein levels across ESCA cell lines and confirmed higher E2F4 expression in TE-1 and KYSE-150 (**Supplementary Figure S4a**). Silencing of E2F4 by two independent shRNAs effectively reduced its protein levels in both cell lines (**Supplementary Figure S4b**). Importantly, E2F4 knockdown did not affect SNRPB2 levels (**Supplementary Figure S4c**), indicating that SNRPB2 acts upstream of E2F4. Moreover, Actinomycin D chase experiments revealed no significant difference in E2F4 mRNA decay following SNRPB2 knockdown (**Supplementary Figure S4d**), suggesting that SNRPB2 does not regulate E2F4 at the transcriptional or mRNA stability level. Together, these results indicate that SNRPB2 promotes E2F4 protein stability by inhibiting proteasomal degradation. Loss of SNRPB2 reduces E2F4 protein levels, thereby relieving its transcriptional repression on cell cycle-associated targets and promoting their upregulation. This mechanism highlights the role of the SNRPB2-E2F4 axis in modulating cell cycle progression in ESCA.

### E2F4 counteracted the inhibition effects of shSNRPB2 on the biology in ESCC

**Figure 5:**
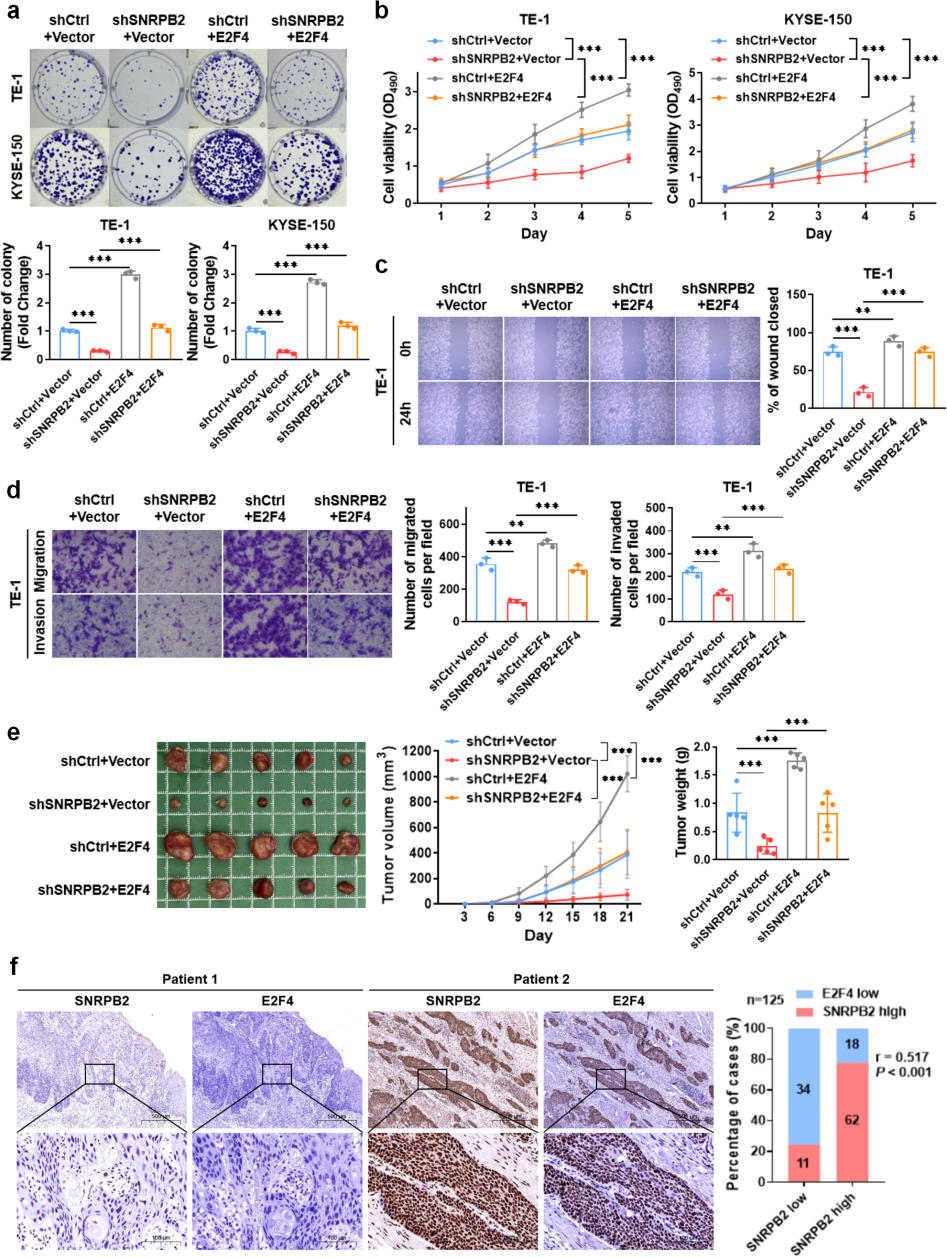
E2F4 overexpression rescued the effects of shSNRPB2 on proliferation, migration and invasion of ESCC cells *in vitro* and *in vivo*. **(a, b)** shCtrl+E2F4 group increased the proliferation and clone formation of ESCC compared with shCtrl+Vector group, and shSNRPB2+E2F4 group also increased the proliferation and clone formation of ESCC compared with shSNRPB2+Vector group. **(c, d)** shCtrl+E2F4 group promoted the migration and invasion of ESCC compared with shCtrl+Vector group, and shSNRPB2+E2F4 group accelerated the migration and invasion of ESCC compared with shSNRPB2+Vector group. **(e)** Five nude mice in each group were subcutaneously injected with TE-1 cells with shCtrl+Vector, shSNRPB2+Vector, shCtrl+E2F4, and shSNRPB2+E2F4. The mean tumor volume (cm^3^) and weight **(g)** were measured three weeks later. **(f)** Representative images of SNRPB2 and E2F4 protein expression in ESCC specimens. Scale bars: upper panels, 500 µm; lower panels, 100 µm. A positive correlation between SNRPB2 and E2F4 expression was observed in 125 ESCC samples. All *in vitro* experiments were performed in triplicate (n = 3); β-actin was used as an internal control where applicable. *In vivo* tumor data represent n = 5 mice per group. Data are presented as mean ± SD. Statistical significance was determined using unpaired Student’s t-test or chi-square test. (**P < 0.01; ***P < 0.001).

To investigate the role of E2F4 in SNRPB2-promoted proliferation, migration, and invasion of ESCC cells, we constructed overexpression plasmid of E2F4 to cotransfect with shSNRPB2 in TE-1 and KYSE-150 cells (**Supplementary Figure S4e**). The results showed that the overexpression of E2F4 (shCtrl+E2F4) group increased cell proliferation and clone formation compared to the shCtrl+Vector group. The shSNRPB2+E2F4 group also showed increased proliferation and clone formation of TE-1 and KYSE-150 cells compared to the shSNRPB2+Vector group (**Figures 5a, b**). In wound healing and transwell assays, overexpression of shCtrl+E2F4 promoted the migration and invasion of TE-1 cells compared to the shCtrl+Vector group, whereas the shSNRPB2+E2F4 group rescued the migration and invasion of TE-1 cells compared to the shSNRPB2+Vector group (**Figures 5c, d**). As shown in **Figures 5e**, E2F4 overexpression also blocked shSNRPB2-decreased ESCC cell progression *in vivo*. These results suggest that E2F4, which is positively regulated by SNRPB2, could be a target gene that plays a role in the progression of ESCC.

Finally, we assessed IHC staining in specimens from 125 patients with ESCC to evaluate whether the SNRPB2-E2F4 axis exists in human ESCC specimens. The results suggested that SNRPB2 expression was significantly positively correlated with E2F4 expression (r = 0.517, *P* < 0.05) (**Figure 5f**, **Table 3**).

**Table 3:** The correlation between SNRPB2 and E2F4 protein expression in esophageal squamous cell carcinoma.

SNRPB2	E2F4		n	R Value	P Value
	Low expression	High expression			
Low expression	34	11	45	0.517	**<0.001**
High expression	18	62	80		
n	52	73	125		

Bold values indicate statistically significant differences (P < 0.05).

## Discussion

Increasing evidence indicates that pre-mRNA splicing contributes to transcriptomic and proteomic diversity, and dysregulation of this process has been implicated in the pathogenesis of numerous diseases, particularly cancers, where splicing factors may serve as biomarkers or therapeutic targets (12, 18). Esophageal squamous cell carcinoma (ESCA) is a biologically heterogeneous malignancy arising through multistep epithelial transformation (19, 20). Improving the poor prognosis of ESCA requires multidisciplinary evaluation and multimodal therapy, centered on identifying robust therapeutic targets and prognostic biomarkers (21).

SNRPB2 is a core component of the spliceosome, essential for 17S U2 snRNP formation and prespliceosome assembly, and its disruption leads to cell death due to failed pre-mRNA splicing (22). It is localized to the nucleus and associated with vimentin-containing intermediate filaments, particularly those surrounding the nucleus (23). Functionally, SNRPB2 participates in posttranscriptional gene regulation and contributes to the functional expansion of the proteome (24). However, its role in cancer biology remains poorly understood. In this study, we conducted a comprehensive analysis of SNRPB2 expression and function, and evaluated its potential as a prognostic biomarker in ESCC.

Our immunohistochemical data showed that SNRPB2 protein levels were significantly elevated in ESCC samples compared to paired normal tissues, in concordance with online datasets. Moreover, high SNRPB2 expression was significantly associated with lymph node metastasis and advanced clinical stage, supporting its potential role in promoting tumor progression (13, 25).

Aberrant gene expression is a hallmark of cancer, often shaped by multiple converging pathways (26, 27). Through GO and GSEA analyses, we found that SNRPB2 inhibits the Rb/E2F pathway, a critical regulator of cell cycle progression. Mechanistically, SNRPB2 stabilizes E2F4 protein, a transcriptional repressor in the Rb/E2F axis (28, 29). Our co-immunoprecipitation and functional assays confirmed that SNRPB2 interacts with E2F4 and prevents its proteasome-mediated degradation, thereby enhancing its stability. Cycloheximide chase and MG132 treatment further validated the proteasome-dependent stabilization of E2F4 by SNRPB2. Notably, silencing SNRPB2 led to de-repression of E2F4 target genes such as *CCNE1*, *PCNA*, *TK1*, and *PLK1*, implicating a direct role in cell cycle regulation. These findings support a non-canonical oncogenic function of SNRPB2 in promoting ESCC progression via the SNRPB2–E2F4 axis.

Although SNRPB2 is classically recognized for its role in pre-mRNA splicing, our current study focuses on its post-translational regulatory functions. We did not assess the transcriptomic consequences of SNRPB2 knockdown on alternative splicing events; however, future studies using RNA-seq or public splicing databases (e.g., TCGA SpliceSeq) are warranted to explore this dimension.

Given that alternative splicing influences immune cell differentiation and activation (30, 31), and considering the increasingly recognized link between RNA splicing and tumor immune modulation, we also explored the immunological relevance of SNRPB2 in ESCA. Bioinformatic analysis revealed that SNRPB2 expression positively correlated with tumor-infiltrating lymphocytes (TILs), MHC molecules, and multiple chemokines. Moreover, SNRPB2 expression was associated with key immune checkpoint molecules such as PD-1, PD-L1, and CTLA-4. qPCR validation confirmed that SNRPB2 knockdown downregulated several immune-regulatory genes, supporting its involvement in immune signaling regulation.

These findings suggest that SNRPB2 may serve as both an oncogene and an immune regulatory factor. Its dual role raises the possibility that SNRPB2 may serve as a biomarker for immunotherapeutic stratification. High SNRPB2 expression may reflect an “immune-inflamed but suppressed” phenotype, which has been associated with enhanced responsiveness to immune checkpoint blockade therapies. Thus, SNRPB2 could be explored not only as a prognostic marker but also as a potential predictive biomarker for immunotherapy response in ESCC. Further clinical studies are needed to validate this possibility.

While our results are promising, several limitations merit discussion. First, our *in vivo* models employed immunodeficient nude mice, limiting the assessment of tumor-immune interactions. Second, only two ESCC cell lines (TE-1 and KYSE-150) were used in functional assays. Although these are widely accepted models for ESCC studies, tumor heterogeneity across different cell lines warrants further exploration. Third, mechanistic insights were largely derived from *in vitro* and subcutaneous xenograft models, which do not fully replicate the complexity of human disease. Orthotopic or patient-derived xenograft models are needed to extend the translational relevance of our findings.

In addition, the discrepancy between mRNA and protein levels of SNRPB2 across datasets and our clinical cohort may reflect tumor heterogeneity, microenvironmental influences, or post-transcriptional regulatory mechanisms. These possibilities should be explored in larger, clinically annotated cohorts to fully understand the regulatory dynamics of SNRPB2.

Future studies will further explore the post-translational mechanisms contributing to E2F4 stabilization by SNRPB2, including the potential roles of deubiquitination and other modifications.

In summary, SNRPB2 is significantly upregulated in ESCC and correlates with tumor progression and poor clinical outcome. It stabilizes E2F4 protein and promotes cell cycle progression, while also modulating immune regulatory pathways. Our findings position SNRPB2 as a potential prognostic and predictive biomarker in ESCC and highlight its potential as a therapeutic target in future intervention strategies.

## Conclusions

SNRPB2 plays a pivotal role in the progression of ESCC by enhancing the stability of E2F4, thereby promoting cell proliferation, migration, and invasion. Moreover, SNRPB2 serves as a significant prognostic marker in ESCA, with potential implications for immunoregulation. Its involvement in these processes underscores its importance as a critical factor for predicting disease outcomes, guiding therapeutic strategies, and facilitating personalized treatment approaches for ESCC patients.

## Data Availability

The original contributions presented in the study are included in the article/**Supplementary Material**. Further inquiries can be directed to the corresponding author.

## Supplementary material

Supplementary Figure 1 SNRPB2 is upregulated in ESCA and associated with aggressive clinical features. **(a)** Pan-cancer analysis of SNRPB2 expression across TCGA tumor and matched normal tissues. Red boxes represent tumor samples; blue boxes represent normal samples. **(b)** Kaplan-Meier curves were used to analyze the influence of SNRPB2 mRNA on PFI in ESCA patients. **(c)** SNRPB2 mRNA expression in ESCC tumor (n = 182) and normal (n = 286) tissues from TCGA dataset. **(d)** Violin plot showed SNRPB2 mRNA expression across different clinical stages in ESCC from TCGA dataset. **(e)** Association of SNRPB2 protein expression with vessel carcinoma embolus and lymph node metastasis in ESCC. **(F)** Representative images of colony formation assays in TE-1 and KYSE-150 cells transfected with control or two independent SNRPB2 shRNAs. Colonies were stained and quantified, corresponding to the statistical analysis in **Figure 2b**. (**P* < 0.05; ****P* < 0.001).

Supplementary Figure 2 Analysis of SNRPB2 genetic alterations in ESCA. **(a)** Genetic alterations were detected in 1.1% of SNRPB2 in ESCA. **(b)** No alteration of SNRPB2 was detected in ESCC.

Supplementary Figure 3 Immunological correlation between SNRPB2 and immune modulatory factors across multiple cancer types. **(a)** Correlation of the expression level of SNRPB2 gene with TILs. **(b, c)** Correlation of the expression level of SNRPB2 gene with multiple immunostimulators and immunoinhibitors. **(d-f)** Correlation of the expression level of SNRPB2 gene with multiple MHC molecules, chemokines and chemokine receptors.

Supplementary Figure 4 SNRPB2 regulates E2F4 protein expression independent of mRNA stability. **(a)** Protein and relative mRNA levels of E2F4 in normal esophageal epithelial cells and ESCC cell lines were determined by western blot and q-PCR, with β-actin as the loading control. Data represent mean ± SD from three independent experiments. **(b)** Western blot analysis of E2F4 protein levels in TE-1 and KYSE-150 cells after transduction with two independent shRNAs targeting E2F4, with β-actin as the loading control. Bar graphs on the right show quantification of relative E2F4 protein levels normalized to β-actin. **(c)** Correlation of the protein expression levels of SNRPB2 and E2F4 in TE-1 and KYSE-150 cells. **(d)** mRNA stability assays using Actinomycin D treatment showed no significant difference in E2F4 mRNA decay between control and SNRPB2 knockdown cells in TE-1 and KYSE-150. **(e)** Overexpression of E2F4 significantly increased its protein levels in both TE-1 and KYSE-150 cells. Quantification is shown in the adjacent bar graphs. β-actin was used as a loading control in all Western blots. Statistical analysis was performed using Student’s t-test and two-sided t-test. (****P* < 0.001; ns, not significant).
