# Supplementary figures and images for: SNRPB2 facilitates esophageal squamous cell carcinoma oncogenesis and progression via E2F4 stabilization
Source: Front Immunol. 2025 Jun 19;16:1610721. doi: 10.3389/fimmu.2025.1610721 (PMC12222229; doi:10.3389/fimmu.2025.1610721)

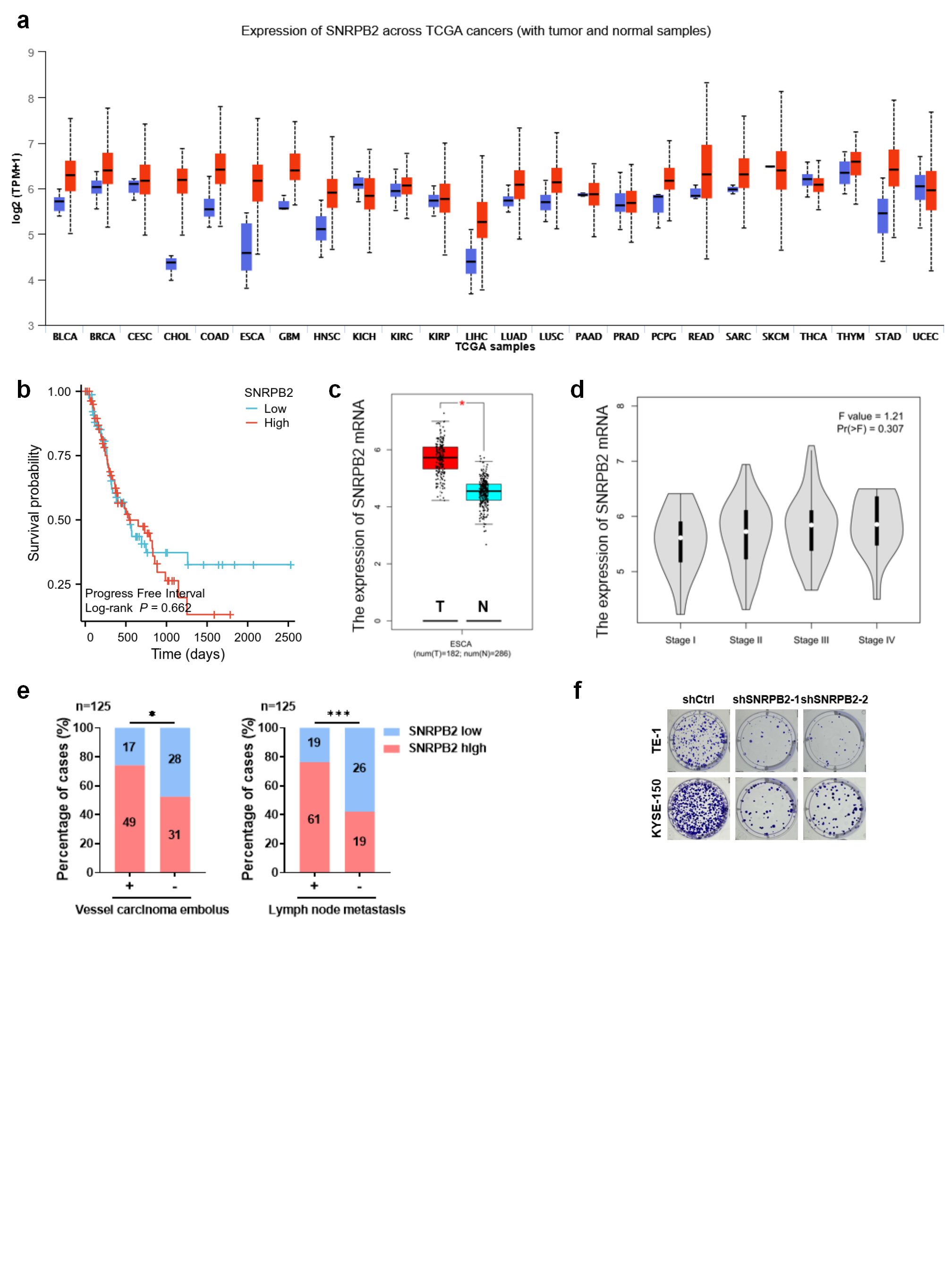

Supplement: Supplementary Figure 1 — SNRPB2 is upregulated in ESCA and associated with aggressive clinical features. (a) Pan-cancer analysis of SNRPB2 expression across TCGA tumor and matched normal tissues. Red boxes represent tumor samples; blue boxes represent normal samples. (b) Kaplan-Meier curves were used to analyze the influence of SNRPB2 mRNA on PFI in ESCA patients. (c) SNRPB2 mRNA expression in ESCC tumor (n = 182) and normal (n = 286) tissues from TCGA dataset. (d) Violin plot showed SNRPB2 mRNA expression across different clinical stages in ESCC from TCGA dataset. (e) Association of SNRPB2 protein expression with vessel carcinoma embolus and lymph node metastasis in ESCC. (F) Representative images of colony formation assays in TE-1 and KYSE-150 cells transfected with control or two independent SNRPB2 shRNAs. Colonies were stained and quantified, corresponding to the statistical analysis in Figure 2b . (*P < 0.05; ***P < 0.001). [file Image1.tif]

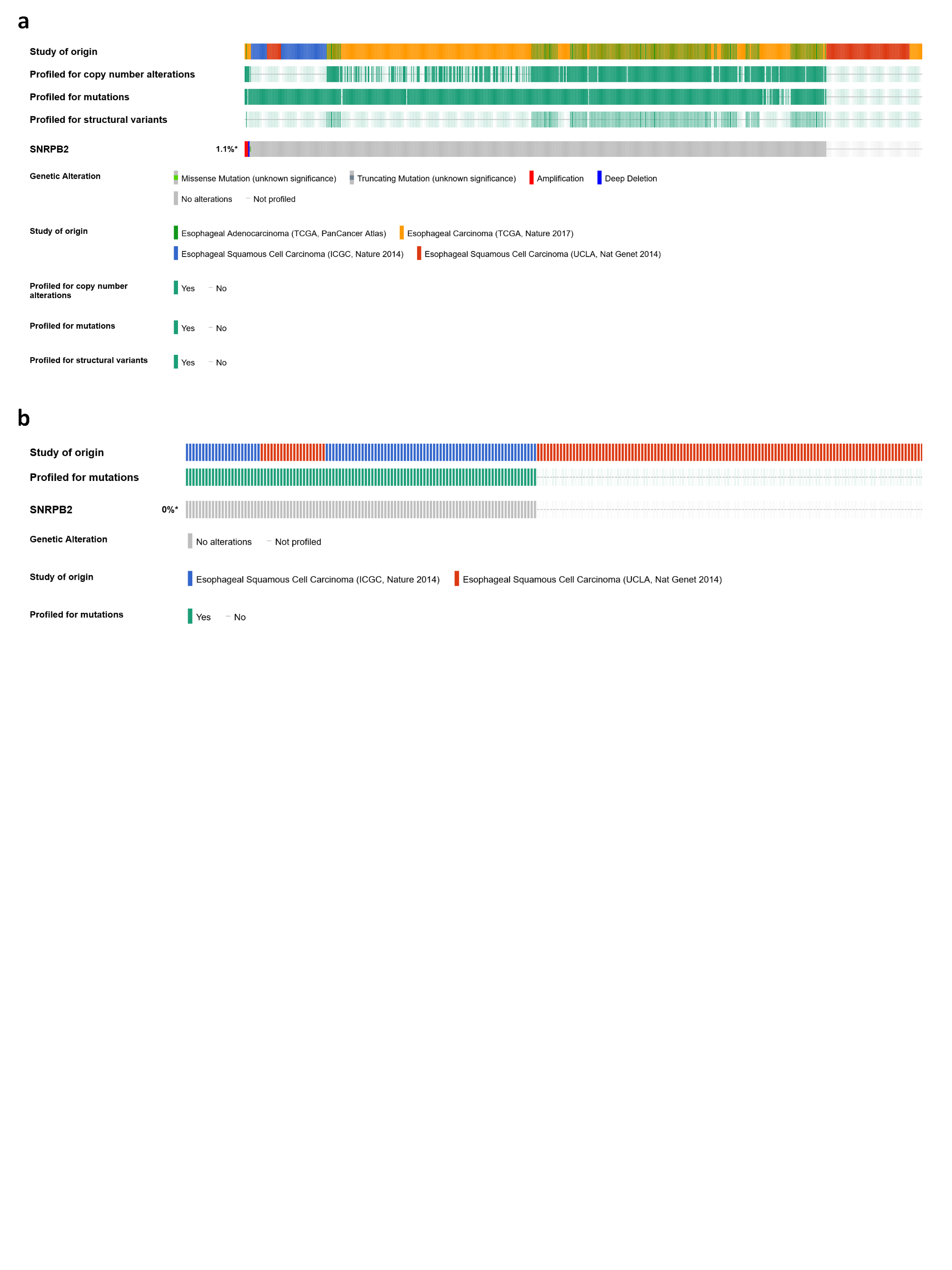

Supplement: Supplementary Figure 2 — Analysis of SNRPB2 genetic alterations in ESCA. (a) Genetic alterations were detected in 1.1% of SNRPB2 in ESCA. (b) No alteration of SNRPB2 was detected in ESCC. [file Image2.tif]

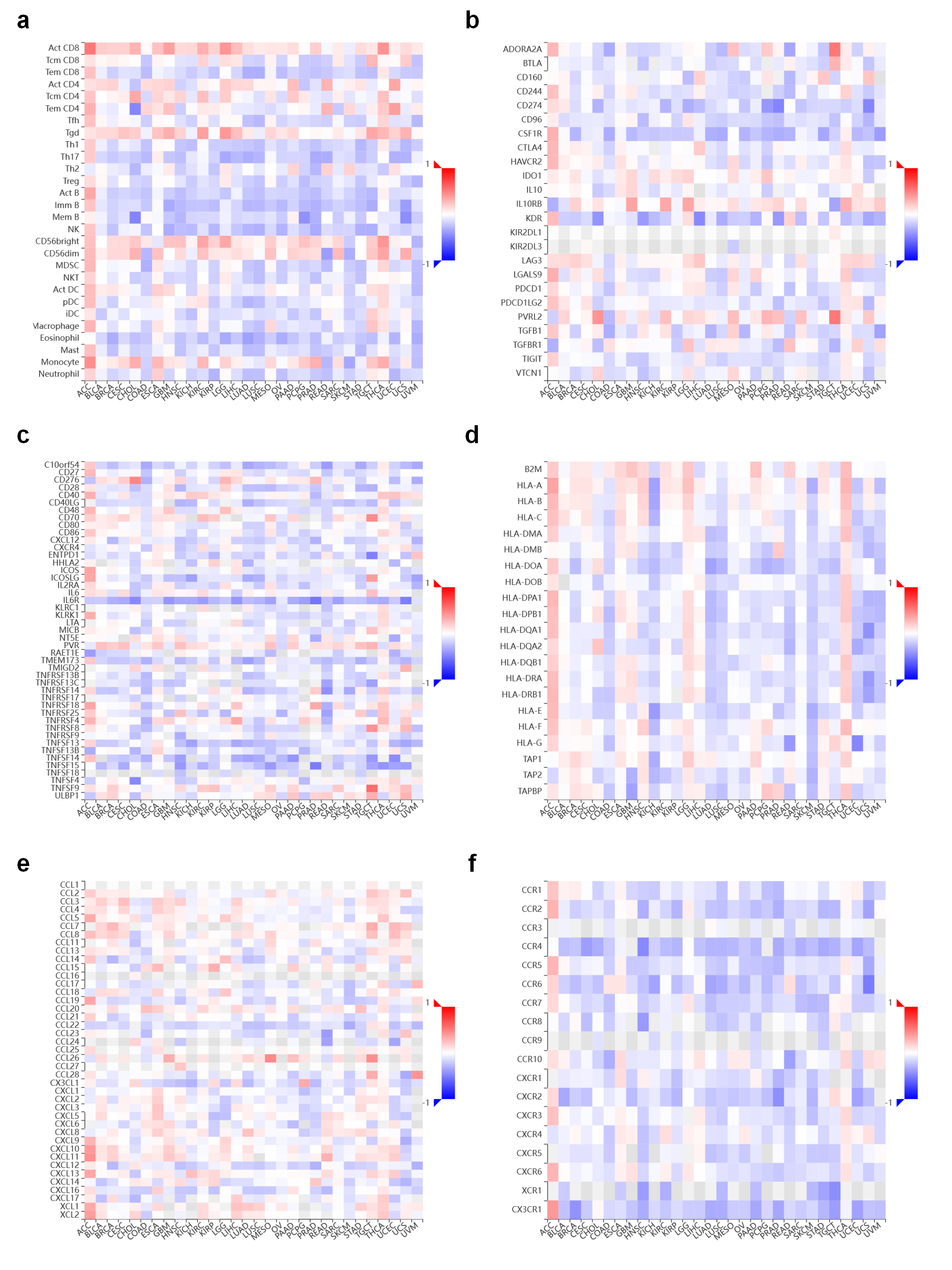

Supplement: Supplementary Figure 3 — Immunological correlation between SNRPB2 and immune modulatory factors across multiple cancer types. (a) Correlation of the expression level of SNRPB2 gene with TILs. (b, c) Correlation of the expression level of SNRPB2 gene with multiple immunostimulators and immunoinhibitors. (d-f) Correlation of the expression level of SNRPB2 gene with multiple MHC molecules, chemokines and chemokine receptors. [file Image3.tif]

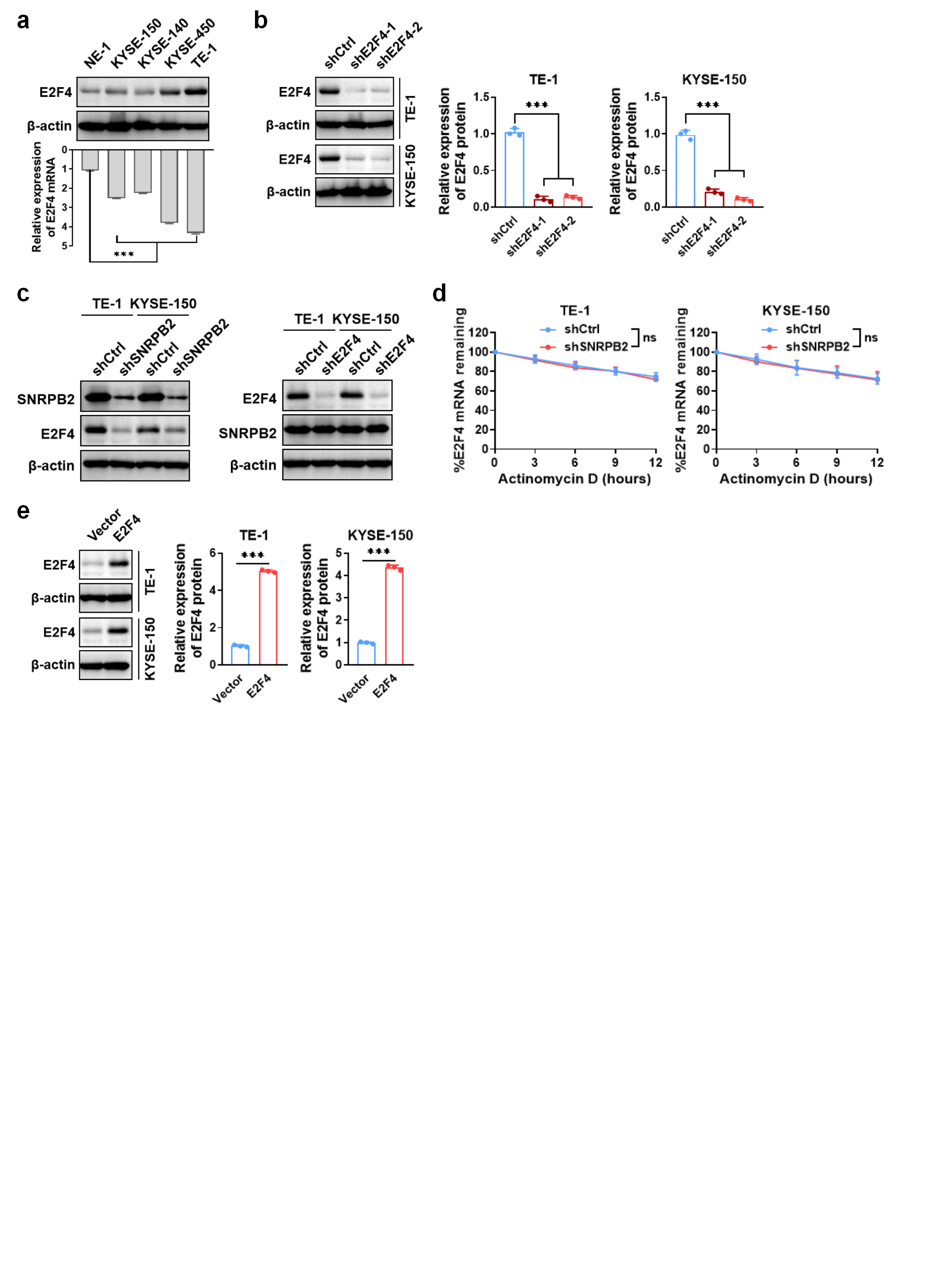

Supplement: Supplementary Figure 4 — SNRPB2 regulates E2F4 protein expression independent of mRNA stability. (a) Protein and relative mRNA levels of E2F4 in normal esophageal epithelial cells and ESCC cell lines were determined by western blot and q-PCR, with β-actin as the loading control. Data represent mean ± SD from three independent experiments. (b) Western blot analysis of E2F4 protein levels in TE-1 and KYSE-150 cells after transduction with two independent shRNAs targeting E2F4, with β-actin as the loading control. Bar graphs on the right show quantification of relative E2F4 protein levels normalized to β-actin. (c) Correlation of the protein expression levels of SNRPB2 and E2F4 in TE-1 and KYSE-150 cells. (d) mRNA stability assays using Actinomycin D treatment showed no significant difference in E2F4 mRNA decay between control and SNRPB2 knockdown cells in TE-1 and KYSE-150. (e) Overexpression of E2F4 significantly increased its protein levels in both TE-1 and KYSE-150 cells. Quantification is shown in the adjacent bar graphs. β-actin was used as a loading control in all Western blots. Statistical analysis was performed using Student’s t-test and two-sided t-test. (***P < 0.001; ns, not significant). [file Image4.tif]
