# Supplementary material for: SNRPB2 facilitates esophageal squamous cell carcinoma oncogenesis and progression via E2F4 stabilization
Source: Front Immunol. 2025 Jun 19;16:1610721. doi: 10.3389/fimmu.2025.1610721 (PMC12222229; doi:10.3389/fimmu.2025.1610721)
Supplement: Supplementary file 5 [file Table1.docx]

| TABLE S1 The correlation with differentially expressed genes of ESCA and clinical stage in GEPIA2 database. | | | | | |
| --- | --- | --- | --- | --- | --- |
| id | gender | stage | T | N | SigNum |
| SNRPB2 | 0.095156346 | 0.015613339 | 0.243766974 | 0.914060647 | 1 |
| NAA10 | 0.609889043 | 0.691153948 | 0.551671368 | 0.686633651 | 0 |
| SMN1 | 0.670229834 | 0.466007183 | 0.098030686 | 0.265315801 | 0 |
| UPF3B | 0.477877172 | 0.419678958 | 0.190530956 | 0.182535538 | 0 |
| BCAP31 | 0.948000745 | 0.450035144 | 0.412031155 | 0.494801485 | 0 |
| YBX2 | 0.25454056 | 0.14583097 | 0.560058894 | 0.452928945 | 0 |
| MIR9-1HG | 0.544414668 | 0.611078541 | 0.768499423 | 0.460165684 | 0 |
| HSPD1 | 0.331751785 | 0.343608199 | 0.395035808 | 0.360941891 | 0 |
| DNAJA1P3 | 0.87199317 | 0.674696744 | 0.629586164 | 0.383565284 | 0 |
| AC026124.2 | 0.66464775 | 0.817474492 | 0.831110983 | 0.383618107 | 0 |
| TMEM270 | 0.832888367 | 0.713148088 | 0.591599438 | 0.211530565 | 0 |
| H2BC8 | 0.727009731 | 0.454018079 | 0.83465804 | 0.319163972 | 0 |
| TERT | 0.367301567 | 0.488069623 | 0.827962462 | 0.658648358 | 0 |
| GLA | 0.66464775 | 0.287659002 | 0.391886257 | 0.360305447 | 0 |
| AC104073.4 | 0.910699332 | 0.908876609 | 0.85946484 | 0.592496184 | 0 |
| VBP1 | 0.881066907 | 0.903479602 | 0.487814762 | 0.519857021 | 0 |
| AL035461.2 | 0.454409318 | 0.299419381 | 0.410452024 | 0.991666314 | 0 |
| AP003696.1 | 0.091413386 | 0.079065268 | 0.299942392 | 0.396630586 | 0 |
