# Supplementary material for: SNRPB2 facilitates esophageal squamous cell carcinoma oncogenesis and progression via E2F4 stabilization
Source: Front Immunol. 2025 Jun 19;16:1610721. doi: 10.3389/fimmu.2025.1610721 (PMC12222229; doi:10.3389/fimmu.2025.1610721)
Supplement: Supplementary file 6 [file Table2.docx]

| TABLE S2 The correlation with differentially expressed genes of ESCA and prognosis in GEPIA2 database. | | | | | |
| --- | --- | --- | --- | --- | --- |
| gene | KM | HR | HR.95L | HR.95H | coxPvalue |
| NAA10 | 0.007911226 | 1.306482378 | 1.148319686 | 1.486429453 | 4.89E-05 |
| FO680682.1 | 0.049099597 | 5.754830882 | 1.129735944 | 29.31488428 | 0.035133487 |
| LARP1P1 | 0.016401766 | 1.67980407 | 1.142991638 | 2.46873347 | 0.008283338 |
| SMN1 | 0.005858289 | 1.418399291 | 1.055905257 | 1.905338132 | 0.020274726 |
| MIR26A2 | 0.022910513 | 0.248637707 | 0.063326346 | 0.97622416 | 0.046103191 |
| UPF3B | 0.004087935 | 1.061131796 | 1.01455654 | 1.109845182 | 0.009569175 |
| ARL5B | 0.005951584 | 1.038419649 | 1.004012363 | 1.074006065 | 0.028315826 |
| BCAP31 | 0.001064296 | 1.025393176 | 1.01322301 | 1.037709523 | 3.85E-05 |
| YBX2 | 0.045886277 | 1.093593139 | 1.026138349 | 1.165482175 | 0.005881836 |
| MIR9-1HG | 0.010731586 | 3.758908965 | 1.428861202 | 9.888571813 | 0.007294012 |
| HSPD1 | 0.015767499 | 1.009825274 | 1.004211483 | 1.015470447 | 0.000587021 |
| DNAJA1P3 | 0.033146583 | 17.14622087 | 1.766068725 | 166.4674121 | 0.01427029 |
| FAM189A2 | 0.016064885 | 0.709475789 | 0.520307423 | 0.967420169 | 0.03005938 |
| SNRPB2 | 0.013223178 | 1.038131587 | 1.016406111 | 1.060321441 | 0.000524332 |
| AC108673.2 | 0.010380326 | 1.452184235 | 1.114699141 | 1.891845947 | 0.00569872 |
| AC026124.2 | 0.005297139 | 3.438678982 | 1.827121808 | 6.471661106 | 0.000129099 |
| AC006460.1 | 0.039951203 | 0.801155131 | 0.65564834 | 0.978953967 | 0.030161321 |
| TMEM270 | 0.017296357 | 1.717801968 | 1.061385146 | 2.780181739 | 0.027631407 |
| H2BC8 | 0.030053014 | 1.042395478 | 1.01010938 | 1.075713536 | 0.009693858 |
| TERT | 0.045059048 | 1.443722493 | 1.114895169 | 1.86953419 | 0.005357632 |
| GLA | 0.026628494 | 1.106262761 | 1.026455632 | 1.192274911 | 0.008206087 |
| AC104073.4 | 0.012734179 | 1.772008923 | 1.050053777 | 2.990337918 | 0.032120829 |
| MIR135B | 0.009249691 | 0.601378039 | 0.40305608 | 0.897283441 | 0.012744265 |
| LINC02811 | 0.022924151 | 1.404056644 | 1.137627644 | 1.73288252 | 0.001572156 |
| CLBA1 | 0.041201458 | 0.735208228 | 0.558595629 | 0.967660881 | 0.028200152 |
| FO393419.3 | 0.009777491 | 2.929992995 | 1.324384342 | 6.482150745 | 0.007967857 |
| VBP1 | 0.003027137 | 1.050740109 | 1.022670553 | 1.079580099 | 0.000340165 |
| AL035461.2 | 0.024327847 | 1.406147787 | 1.10450488 | 1.790170088 | 0.005661029 |
| KRI1 | 0.000623481 | 0.884469972 | 0.799118492 | 0.97893759 | 0.017734898 |
| LEMD1 | 0.017435446 | 0.870810768 | 0.763409961 | 0.993321326 | 0.039423112 |
| AP003696.1 | 0.000856668 | 1.725515359 | 1.15761509 | 2.572014895 | 0.007392479 |
